# Current Landscape and the Potential Role of Hypoxia-Inducible Factors and Selenium in Clear Cell Renal Cell Carcinoma Treatment

**DOI:** 10.3390/ijms19123834

**Published:** 2018-12-01

**Authors:** Rohan Garje, Josiah J. An, Kevin Sanchez, Austin Greco, Jeffrey Stolwijk, Eric Devor, Youcef Rustum, Yousef Zakharia

**Affiliations:** 1 Department of Internal Medicine, Division of Hematology and Oncology, University of Iowa, Iowa City, IA 52242, USA; rohan-garje@uiowa.edu; josiah-an@uiowa.edu (R.G.); josiah-an@uiowa.edu (J.J.A.); Youcef.Rustum@RoswellPark.org (Y.R.); 2 Holden Comprehensive Cancer Center, University of Iowa, Iowa City, IA 52242, USA; 3 Department of Internal Medicine, University of Iowa, Iowa City, IA 52242, USA; kevin-sanchez@uiowa.edu (K.S.); austin-greco@uiowa.edu (A.G.); 4 Interdisciplinary Graduate Program in Human Toxicology, Department of Occupational and Environmental Health, College of Public Health, University of Iowa, Iowa City, IA 52242, USA; jeffrey-stolwijk@uiowa.edu; 5 Department of Obstetrics and Gynecology, University of Iowa, Iowa City, IA 52242, USA; eric-devor@uiowa.edu; 6 Roswell Park Cancer Institute, Buffalo, NY 14203, USA

**Keywords:** clear cell renal cell carcinoma, hypoxia-inducible factors (HIFs), selenium, PD-L1, miRNA, VEGF, mTOR inhibitors

## Abstract

In the last two decades, the discovery of various pathways involved in renal cell carcinoma (RCC) has led to the development of biologically-driven targeted therapies. Hypoxia-inducible factors (HIFs), angiogenic growth factors, von Hippel–Lindau (*VHL*) gene mutations, and oncogenic microRNAs (miRNAs) play essential roles in the pathogenesis and drug resistance of clear cell renal cell carcinoma. These insights have led to the development of vascular endothelial growth factor (VEGF) inhibitors, Mechanistic target of rapamycin (mTOR) inhibitors, and immunotherapeutic agents, which have significantly improved the outcomes of patients with advanced RCC. HIF inhibitors will be a valuable asset in the growing therapeutic armamentarium of RCC. Various histone deacetylase (HDAC) inhibitors, selenium, and agents like PT2385 and PT2977 are being explored in various clinical trials as potential HIF inhibitors, to ameliorate the outcomes of RCC patients. In this article, we will review the current treatment options and highlight the potential role of selenium in the modulation of drug resistance biomarkers expressed in clear cell RCC (ccRCC) tumors.

## 1. Introduction

Clear cell renal cell carcinoma (ccRCC) is the most common malignancy in the kidney. Over 65,000 new kidney cancer cases and 14,000 deaths were estimated in the United States in 2018 [1]. Renal cell carcinoma (RCC) is the most lethal genitourinary cancer, given that its disease course is largely asymptomatic and incidentally found in more than half of new cases [2,3]. Established modifiable risk factors for RCC include obesity, smoking, and hypertension [4]. Other studies link alcohol use, type 2 diabetes, and occupational or environmental exposures to increased risk of RCC [5].

RCC is categorized into three major histological subtypes: ccRCC, comprising 70% of cases; papillary and chromophobe RCC, which together comprise 25% of cases; and tumors of the medullary and collecting systems, which comprise 5% of cases [6,7]. These subtypes arise from distinct genetics and therefore are treated differently [8].

Localized RCC is often managed surgically with a partial or radical nephrectomy, with tumor ablation or active surveillance for small tumors. Systemic therapy is primarily reserved for metastatic RCC. Current evidence for adjuvant systemic therapy after complete resection of the tumor has shown no survival benefit [9]. For stage IV disease, cytoreductive nephrectomy in addition to systemic therapy has not shown improvement in overall survival [9,10]. In the last two decades, there has been significant improvement in our knowledge of renal cell carcinogenesis that has, in turn, led to the development of biologically-driven targeted therapies.

## 2. Role of Hypoxia-Inducible Factors in Renal Cell Carcinogenesis

**Figure 1:**
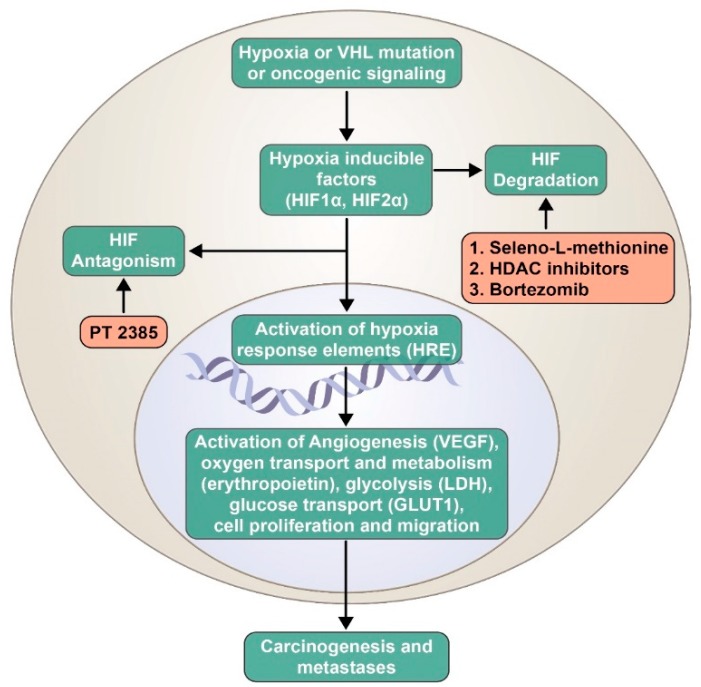
Inhibitors of the hypoxia-inducible factor (HIF) pathway currently being evaluated in clinical trials. VHL: von Hippel–Lindau; HIF: hypoxia-inducible factors; HDAC: histone deacetylase; VEGF: vascular endothelial growth factor; LDH: lactate dehydrogenase; GLUT1: glucose transporter 1.

Adaptation to a hypoxic environment is a key attribute of cancer cells. This is mediated via transcription factors called hypoxia-inducible factors (HIFs). These factors are heterodimers with an α-subunit (HIF1α, HIF2α, or HIF3α) and a β-subunit (HIF1β) [11]. Previously, HIF1α was considered to be a predominant oncogenic driver, but recent evidence shows HIF2α as a key player in renal cancer progression [12]. Along with hypoxia, the von Hippel–Lindau (*VHL*) gene and other oncogenic signaling pathways (e.g., PI3K, RAS) are known to regulate HIF activation. Once activated, HIF transcription factors translocate to the nucleus and bind to the hypoxia response elements, which leads to transcription of several target genes involved in angiogenesis (vascular endothelial growth factor (VEGF)), oxygen transport and metabolism (erythropoietin), glycolysis (LDH), glucose transport (GLUT1), cell proliferation, and migration, which eventually leads to carcinogenesis (Figure 1) [13,14]. VEGF plays a vital role in tumor angiogenesis, and is a key target of anti-cancer therapeutic agents. Regulation of the HIF pathway is vital for cellular homeostasis.

## 3. Regulation of HIF Pathway by VHL Gene

Von Hippel–Lindau (*VHL*) is a tumor suppressor gene located on the short arm of chromosome 3 that is commonly mutated in both hereditary and sporadic renal cell carcinoma. The *VHL* gene encodes two isoforms of VHL proteins (pVHL) that play a crucial role in cellular oxygen sensing and regulation of HIFs. In normoxic conditions, the pVHL form a ubiquitin ligase complex and bind to the hydroxylated HIF1α and HIF2α, which subsequently undergoes ubiquitination and proteasomal degradation. However, in cellular hypoxic conditions, the pVHL ubiquitin ligase complex cannot bind to HIFα and lead to its degradation, as they are not hydroxylated (an oxygen-dependent process). Hence, this leads to HIFα accumulation and formation of heterodimer complex with HIF1β and subsequent induction of several target genes in the nucleus [15,16]. In ccRCC, a wide range of intragenic mutations, deletions (complete or partial) and splicing defects have been identified that derange normal function of the *VHL* gene, which creates a situation similar to cellular hypoxia and the accumulation of HIFs [17].

In addition to the *VHL* gene, multitudinous genetic and enzymatic derangements have been identified that predispose a region to various histologies of renal cell carcinoma. These include folliculin (*FLCN*; chromophobe RCC/oncocytoma in Birt–Hogg–Dubé syndrome), papillary type 1 RCC (*MET*), fumarate hydratase (FH; papillary type 2 RCC in hereditary leiomyomatosis and renal cell cancer syndrome), SDHB/SDHD/SDHC/SDHA (succinate dehydrogenase subunit-related RCC), chromosome 3 translocations-associated clear cell RCC, papillary RCC (PTEN), and BAP1 (clear cell RCC) [18].

This interdependency on biological pathways by cancer cells has laid the foundation for the development of several targeted therapeutic agents for the treatment of advanced renal cell carcinoma.

## 4. Angiogenesis (Vascular Endothelial Growth Factor Pathway) Inhibitors

Current first-line therapy for stage IV, unresectable, or relapsed disease of clear cell histology includes the oral VEGF tyrosine kinase inhibitors (TKIs) sunitinib and pazopanib [9]. Additionally, for intermediate to poor risk groups, based on the international metastatic renal cell carcinoma database consortium (IMDC) criteria [19], either the combination of ipilimumab and nivolumab or cabozantinib are options.

Sunitinib is a multi-kinase inhibitor targeting several tyrosine kinase receptors, including platelet-derived growth factor receptors (PDGFR-α and -β), VEGF receptors (VEGFR-1, -2, and -3), stem cell factor receptor (c-KIT), FMS-like tyrosine kinase (FLT-3), colony-stimulating factor (CSF-1R), and neurotropic factor receptor (RET) [9]. In the landmark phase III, multicenter clinical trial by Motzer et al., sunitinib was compared with interferon-α in patients with previously untreated metastatic renal-cell carcinoma [20]. Progression-free survival (PFS) in the sunitinib arm was 11 months, and in the interferon-α arm the PFS was 5 months. The overall survival (OS) with sunitinib was 26 months.

Pazopanib is another oral angiogenesis inhibitor targeting VEGFR-1, -2, and -3, PDGFR-α and -β, and c-KIT. In a phase III, open-label study of pazopanib in patients with no prior treatment or one prior cytokine-based treatment, PFS was prolonged significantly with pazopanib versus a placebo. For the treatment naïve group, PFS was 11.1 months, compared to 2.8 months for pazopanib and placebo, respectively [21]. In a phase III non-inferiority trial, pazopanib was compared to sunitinib in patients with advanced renal cell carcinoma. The study was positive for non-inferiority, with a progression-free survival of 8.4 and 9.5 months for pazopanib and sunitinib, respectively [22]. In addition, the median OS with pazopanib was 28.3 and 29.1 months for sunitinib. In subgroup analysis for patients with favorable-risk disease, the median OS for pazopanib and sunitinib was found to be 52.5 and 43.6 months, respectively [23]. Both of these medications had similar rates of adverse events that led to dose reduction, and had no differences in grades 3/4 adverse events. Symptoms associated with discomfort, such as fatigue, hand–foot syndrome, and mouth sores occurred more frequently with sunitinib, while pazopanib was associated with elevations in liver-function tests, weight loss, and changes in hair color. The study also showed lower monthly use of medical resources with pazopanib than with sunitinib [22].

Cabozantinib is a small molecule inhibitor of tyrosine kinases, which include VEGF receptors, MET, and AXL [9]. Cabozantinib was compared to sunitinib in a phase II study of intermediate to poor IMDC risk, treatment naïve patients with metastatic RCC [24]. In this study, PFS was 8.6 months versus 5.3 months for cabozantinib and sunitinib, respectively, and the median OS was found to be 34.5 months and 26.6 months, respectively. Based on these results, cabozantinib has been approved by the United States Food and Drug Administration (FDA) as a first-line agent. Cabozantinib has also been studied in a phase III trial (METEOR trial) of patients with disease progression after previous TKI therapy [25]. The study compared second-line therapy with cabozantinib versus everolimus. The results showed a median PFS of 7.4 months compared to 3.8 months for cabozantinib and everolimus, respectively. Thus, in addition to first-line therapy, cabozantinib is a viable option as a second-line therapy for patients with disease progression after other TKI therapy.

Axitinib is a selective, second-generation tyrosine kinase inhibitor targeting VEGFR-1, -2, and -3 [9]. The phase III AXIS trial compared axitinib and sorafenib as second-line therapy, following other systemic therapy. PFS was 6.7 for axitinib versus 4.7 months for sorafenib. PFS was favored in both subgroups of patients treated with axitinib whose prior systemic therapy was sunitinib or cytokine therapy. Median OS was 20.1 months with axitinib, as compared to 19.2 months with sorafenib, although this was not statistically significant [26].

Bevacizumab along with interferon (IFN) α-2b also has a category 1 level of evidence for first-line therapy. It is a recombinant humanized monoclonal antibody that binds to circulating VEGF-A. A double-blind phase III trial (AVOREN) compared bevacizumab plus IFN-α-2b versus placebo plus IFN-α-2b [27]. With the addition of bevacizumab, PFS was significantly increased (10.4 versus 5.4 months), with a tumor response rate of 30.6% in the bevacizumab group compared to 12.4% in the placebo group. This was achieved without significant increase in adverse events. OS was improved in the bevacizumab group versus the placebo group (23.3 versus 21.3 months); however, this was not statistically significant.

## 5. Mechanistic Target of Rapamycin Inhibitors

Mechanistic target of rapamycin (mTOR) proteins are known to regulate cellular metabolism, growth, apoptosis, and angiogenesis through protein expression. Cellular growth factors stimulate the PI3K/Akt/mTOR pathway and eventually lead to HIF accumulation [28]. These discoveries led to the evaluation of temsirolimus and everolimus, which are both mTOR inhibitors in the management of renal cell carcinoma. Temsirolimus was compared to interferon-α in previously untreated patients with poor risk prognostic risk factors per, the MSKCC prognostic model [29]. The group receiving temsirolimus alone demonstrated significant improvement in median OS compared to IFN-α alone (10.9 versus 7.3 months, respectively). Similarly, PFS was shown to have improved from 3.1 months with IFN-α to 5.5 months with temsirolimus. Based on these study results, temsirolimus was the first FDA-approved mTOR inhibitor for patients with advanced renal cell carcinoma [30]. Currently, temsirolimus is the only mTOR inhibitor that is FDA-approved as a monotherapy.

Everolimus in combination with lenvatinib, a TKI, is utilized in patients who progress after prior therapy. In a phase II clinical trial, lenvatinib plus everolimus was compared to single-agent everolimus in previously-treated metastatic RCC (mRCC) patients [31]. The combination therapy showed increased median OS of 25.5 months, compared to 15.4 months for the monotherapy. Similarly, median PFS improved to 14.6 months in the combination group, compared to 5.5 months for the everolimus-only group [31].

## 6. Immunotherapy

Until late 2005, medical treatment options for RCC involved cytokine-based immunotherapy with the use of high-dose interleukin-2 (IL-2) and IFN-α. Though high-dose IL-2 is associated with significant toxicity, long-term durable response rates were seen in a small fraction of patients. High-dose IL-2 therapy is utilized in highly selected patients with excellent performance status and normal organ function [32]. IFN-α as a monotherapy has fallen out of favor, as a phase III multinational trial between sunitinib and IFN-α demonstrated a strong trend toward a median overall survival advantage of sunitinib over IFN-α [33].

Checkmate-214, a randomized phase III clinical trial, evaluated the combination of two immune checkpoint inhibitors, nivolumab and ipilimumab, in comparison to sunitinib in treatment-naïve patients with metastatic ccRCC. In patients with IMDC intermediate and poor risk, PFS was found to be 11.6 and 8.4 months for the nivolumab/ipilimumab combination and sunitinib, respectively. However, discontinuation due to adverse events was 24% in the combination group, as compared to 12% in the sunitinib group. The median OS with sunitinib was 26 months, whereas the median OS was not reached with the combination therapy [34]. Nivolumab was also shown to be effective as second-line therapy. In a phase III trial that studied patients previously treated with at least one line of therapy excluding mTOR inhibitors, nivolumab demonstrated an increase in OS of 5.4 months in comparison to everolimus monotherapy (25 versus 19.6 months, respectively). Median PFS, however, was not statistically significant, with 4.6 months for nivolumab and 4.4 months for everolimus [35].

## 7. Strategies to Inhibit the Hypoxia-Inducible Factor Pathway: A Plausible Therapeutic Avenue

VEGF inhibitors target one of the myriad oncogenic pathways that are activated by HIF. Hence, the cancer eventually develops resistance and progresses, despite an initial good response to various oral TKIs. Inhibiting the HIF pathway and subsequently its translational activity is an attractive treatment modality, as it blocks the activation of all downstream genes. The mTOR inhibitors inhibit HIF activation, but the responses are limited, as noted above. Recently, further strategies have been explored targeting the HIF pathway in combination with VEGF inhibitors, with variable success.

HIF regulation, either by blocking its production, antagonizing it effects, or by enhancing its degradation, has provided multiple opportunities to expand the current therapeutic armamentarium of renal cell carcinoma. In a small study of mRCC, HIF expression was predictive of increased response to sunitinib treatment [36]. In this study, 26 of 49 patients had high HIF1α and HIF2α expression on the tumor cells (based on immunoblot analysis). These patients had a higher rate of complete or partial response when compared to patients with low or absent HIF1α/HIF2α expression.

## 8. Role of Selenium in Cancer Therapeutics and the Hypoxia-Inducible Factor Pathway

Selenium is an essential micronutrient; in the human body, it is involved with the regulation of cell metabolism, DNA, and RNA, as well as protein synthesis, and is at the active site of several enzymes of the antioxidant network [37]. Inorganic forms of selenium, such as selenide and selenite, are converted by plants into organic forms, such as selenomethionine (SLM) and Se-methylselenocysteine (MSC), which are retained in the human body [37]. Epidemiologic studies have suggested that dietary selenium intake is a protective factor for some forms of cancers, such as colorectal, prostate, lung, and bladder cancer [38,39]. However, additional studies in healthy men did not show benefit of selenium in the prevention of prostate cancer [40].

SLM and MSC are forms of selenium that are currently being investigated as possible anti-tumor agents. In their natural form, these agents have a relatively low toxicity profile. They are converted via β-lyase into the active form methylselenol (MSA). HIF1α appears to be a target of selenium. In pre-clinical studies of head and neck squamous cell carcinoma cells that express HIF1α, it was found that in the setting of hypoxia, where HIF1α expression is increased, the cytotoxicity of SN38, the active metabolite of irinotecan, was enhanced with the addition of MSA [41]. This is possibly due to the inhibition of HIF1α by MSA, and demonstrates the potential for reversal of chemoresistance by MSA. Moreover, selenium has been found to target β-catenin, and increases drug cytotoxicity through the reduction of β-catenin’s drug-resistant effects [42]. Selenium compounds may also improve efficaciousness of other anti-tumor agents through a reduction in treatment-induced toxicities, allowing for higher tolerated doses. In one study of A253 and HT 29 xenografts, coadministration of MSC with irinotecan at two to three times the maximum tolerated dose of irinotecan led to a response without intolerable toxicity [43].

Selenium can also affect the tumor microenvironment (TME), and may be able to stabilize the TME to improve drug delivery. MSC has an anti-vascular effect, and can increase the antitumor effect of irinotecan through the inhibition of HIF1α, which leads to decreased microvessel density, lowered tumor interstitial pressure, and increased pericyte coverage of blood vessels [41].

Selenium may also be able to act through its effects in the expression of miRNAs. Non-coding miRNAs are small molecules involved in post-transcriptional regulation of genes, which are often associated with increased angiogenesis and drug resistance.

## 9. Base-Line Transcription and Translation Biomarkers Expressed in Clear Cell Renal Cell Carcinoma Cell Lines with Disentail Expression of HIF1α and HIF2α

The expression levels of oncogenic, tumor-suppressor miRNAs, as well as hypoxia-inducible factors 1α and 2α, and program death ligand1 (PD-L1), are altered in many advanced cancers and implicated in multi-drug resistance, angiogenesis, and tumor growth and metastasis. Specifically, the oncogenic miRNA-155 and miRNA-210 are highly expressed in ccRCC tumors with differential expression of HIFs [44,45].

**Figure 2:**
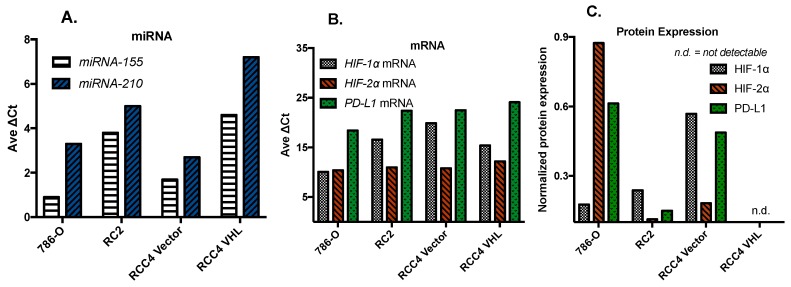
Base-line levels of constitutively expressed miRNAs (**A**), mRNAs (**B**), and HIFs and PD-L1 proteins (**C**) in normoxic clear cell renal cell carcinoma (ccRCC) cell lines. These cell lines show minimal differences in mRNA levels of *PD-L1*, *HIF-1α*, and *HIF-2α* (**B**), but express differential levels of the PD-L1, HIF-1α, and HIF-2α proteins (**C**). Ave ∆*C*t: average cycle threshold change compared to the reference sequence.

We demonstrated that these miRNAs and HIF proteins are targets of therapeutic doses and a schedule of selenomethionine and methylselenocysteine (selenium). It was not fully understood, however, whether selenium exerts its effects at the transcription or at the translation levels. Studies were carried out in ccRCC cell lines expressing differential levels of HIFs. We have determined that the base-line expression of three genes and the two oncogenic miRNAs in four cell lines: 786-O, RC2, RCC4, and RCC4-VHL. The three genes are *HIF-1α*, *HIF-2α*, and *PD-L1*. The two miRNAs are hsa-miR-155 and hsa-miR-210, Figure 2A. Results shown in Figure 2B indicate that there is robust mRNA transcription of each locus in all four of the cell lines. Note that lower normalized transcription levels (∆*C*t) indicate higher expression levels. In general, the 786-O cell line expressing *HIF2α* displays the highest transcription. However, translation of these messages into a protein, as shown in Figure 2C, reveals a very different pattern. For example, in the 786-O cell line, both *HIF-1α* and *HIF-2α* are equally expressed at the RNA level, but reveal very different protein levels. Similarly, the RC2 cell line has consistently lower levels of RNA transcripts than 786-O, but higher levels of protein for *HIF-2α* and *PD-L1*. These inconsistencies suggest that there may be post-transcriptional targeting of these mRNAs by the miRNAs or another, as yet unknown post-transcriptional process at work.

In normal cells, selenium has been shown to have a selective protective effect against chemotherapy-induced DNA damage via p53 mediated DNA repair. However, it did not confer a similar benefit to cancer cells [46].

SLM has been FDA-approved for clinical trials. A phase Ib dose-escalation trial in patients with metastatic ccRCC after failure of prior treatment is ongoing (NCT02535533). Preliminary results of nine evaluable patients demonstrated two patients achieving complete response, and three patients achieving partial response. No dose-limiting toxicities have been noted. The most common side effects included anorexia, fatigue, cough, diarrhea, and proteinuria. There were no grade 4 toxicities or deaths associated with this combination therapy. The phase II part of the clinical trial with SLM and axitinib 5 mg twice daily is planned [47]. The multiple avenues in which selenium interacts with other chemotherapies, the tumor microenvironment, and its interaction with miRNA and transcription factors make it a very favorable target for further research.

## 10. Studies on Hypoxia-Inducible Factor Inhibitors in Advanced Renal Cell Carcinoma

PT2385 is an HIF-2α antagonist that was evaluated in a phase I, standard “3 + 3” dose escalation study of heavily pretreated metastatic ccRCC [48]. In this study, 51 patients were treated with oral PT2385 twice a day, and the recommended phase-II dose (RP2D) was 800 mg BID. One patient had complete response (2%), six had partial responses (12%), and the rest had either stable disease or progression. No dose-limiting toxicities were noted. The most common treatment-related side effects included anemia, peripheral edema, fatigue, and nausea. Considering the promising response signals of a single agent in a heavily pretreated patient population, further studies are ongoing, with a combination of PT2385 with nivolumab and cabozantinib, respectively (NCT02293980).

In a multi-institutional, phase I/II clinical trial, vorinostat (histone deacetylase inhibitor) was evaluated in combination with bevacizumab in ccRCC patients [49]. HDAC inhibitors modulate the HIF pathway by affecting Hsp90 acetylation and HIF-α nuclear translocation [50,51]. In this study, 33 evaluable patients were treated with vorinostat 200 mg orally, twice daily for two weeks, in combination with bevacizumab 15 mg/kg administered intravenously every three weeks. There were no dose-limiting toxicities. Two patients had grade 4 thrombocytopenia. The most common adverse events included fatigue, nausea, pain, anorexia, diarrhea, and elevated creatinine. About 10 patients discontinued therapy due to toxicities, but there were no treatment-related deaths. One patient achieved complete response, and five patients had partial responses. Currently, a phase I/Ib study of pembrolizumab with vorinostat is in progress for patients with advanced renal or urothelial cell carcinoma (NCT02619253).

The safety and efficacy of another HDAC inhibitor, abexinostat, as an epigenetic downregulator of HIF-1α and VEGF expression was evaluated in combination with pazopanib by Aggarwal and colleagues, in a study of advanced solid tumor malignancies [52]. The RCC cohort included 22 patients. The dosing schedule of abexinostat was modified, due to five dose-limiting toxicities that included grade 3 thrombocytopenia (*n* = 2), grade 3 fatigue (*n* = 2), and grade 3 AST/ALT elevation (*n* = 1). There were no treatment-related deaths. The objective response rate in the RCC cohort was 27%, including patients who were previously refractory to pazopanib.

Bortezomib is a proteasome inhibitor currently approved for the treatment of multiple myeloma and mantle cell lymphoma. It is a reversible inhibitor of the chymotrypsin-like activity of the 26S proteasome in mammalian cells. By inhibiting proteasomes, it causes protein buildup and then leads to cell cytotoxicity. In preclinical models, Shin and colleagues have shown its role in *HIF-1α* repression by inhibiting the recruitment of the p300 coactivator [53]. In a phase II clinical trial of treatment-naïve, metastatic ccRCC, 17 patients were treated with sorafenib 200 mg orally twice daily, in combination with bortezomib 1 mg/m^2^ intravenously administered on days 1, 4, 8, and 11, and then every 21 days [54]. The combination was safe, but the study was negative, as it did not meet the pre-specified endpoint of median progression-free survival of 70 weeks. Further studies are not planned with this combination.

The clinical efficacy of bortezomib in combination with bevacizumab was evaluated in 91 patients with treatment-refractory advanced cancers [55]. In the RCC cohort, 5 of 20 patients had a partial response or stable disease. No treatment-related deaths were noted. Common toxicities included thrombocytopenia, fatigue, nausea/vomiting, diarrhea, neuropathy, anemia, neutropenia, and hypertension. Table 1 summarizes the concluded clinical trials.

## 11. Conclusions

Insights into the molecular pathogenesis of ccRCC, especially the HIF pathway, have led to discovery of several therapeutic agents that have improved the treatment landscape. However, new strategies, which are durable and eventually a step closer to potential cure, are needed to further improve responses. HIF inhibition, either as monotherapy or in combination with other VEGF inhibitors, mTOR inhibitors, or immunotherapeutic agents, is promising. Numerous studies are underway evaluating these potentially synergistic combinations. (See Table 2). The preliminary results of SLM in early phase clinical trials of ccRCC are encouraging.

## Figures and Tables

**Table 1:** Summary of reported clinical trials exploring HIF inhibitors in metastatic renal cell carcinoma.

Investigational Agent(s)	Phase	*N*	Trial Design	Dose-Limiting Toxicities (DLTs)	ORR	PFS, OS
**HIF Antagonist**						
PT2385	Phase 1	51	PT2385 administered twice daily orally from 100 to 1800 mg, followed by RP2D expansion phase	No DLTs reported	CR: 2% PR: 12% SD: 52% PD: 34%	PFS, OS: N/A
**HIF Degradation**						
Seleno-L-methionine (SLM) + axitinib	Phase 1b	9	SLM administered at 2500, 3000, or 4000 µg twice daily orally for 14 days, followed by once daily in combination with axitinib	No DLTs reported	CR: 22% PR: 33% SD: 11% PD: 33%	PFS, OS: N/A
**HIF Degradation via Proteasomes**						
Vorinostat + bevacizumab	Phase 1/2	36	Vorinostat administered at 200 mg twice daily orally for 14 days, in combination with bevacizumab at 15 mg/kg intravenously every 3 weeks	No DLTs reported in phase 1; 2 patients with grade 4 thrombocytopenia and grade 3 thromboembolic events	CR: 2.7% PR: 13.8%	mPFS: 5.7 months mOS: 12.9 months
Abexinostat + pazopanib	Phase 1	RCC cohort: 22 Total: 51	Pazopanib administered once daily on days 1 to 28, and abexinostat orally twice daily on days 1 to 5, 8 to 12, and 15 to 19, or on days 1 to 4, 8 to 11, and 15 to 18	Total cohort: 5 DLTs were reported, including fatigue in 2 patients, thrombocytopenia in 2 patients, and elevated transaminases in 1 patient	RCC cohort: ORR (CR, PR): 27%	PFS: N/A OS: N/A
Bortezomib + bevacizumab	Phase 1	91	Bevacizumab administered at 2.5–15 mg/kg intravenously on day 1 of each 21 day cycle; bortezomib administered at 0.7–1.3 mg/m^2^ intravenously on days 1, 4, 8, and 11 of each 21 day cycle	One patient with DLT from acute renal failure at highest dose level; 4 patients with partial response, 7 patients with stable disease at 6 months; toxicities included thrombocytopenia in 23% and fatigue in 19% of patients	CR: 0% PR: 4.4% SD: 42% PD: 47%	PFS: N/A OS: N/A
Sorafenib + bortezomib	Phase 2	17	Sorafenib administered orally twice daily in combination with bortezomib 1 mg/m^2^ intravenously on days 1, 4, 8, and 11, then every 21 days	N/A	CR: 0% PR: 5.8% SD: 70% PD: 23%	mPFS: 13.7 weeks mOS: 110 weeks

CR: complete response; PR: partial response; SD: stable disease; PD: progressive disease; ORR: objective response rate; OS: overall survival; mOS: median overall survival; N/A: not available; HIF: hypoxia-inducible factor; RP2D: recommended phase II dose; RCC: renal cell carcinoma; PFS: Progression-free survival; mPFS: median progression free survival.

**Table 2:** Ongoing clinical trials of HIF inhibitors in metastatic renal cell carcinoma.

Clinicaltrials.gov NCT Identification Number	Phase	Title	*N*	Allocation/Treatment	Primary Objective/Outcome Measures	Status	Expected Completion
NCT03401788	Phase 2	A Phase 2 Study of PT2977 for the Treatment of Von Hippel-Lindau Disease-Associated Renal Cell Carcinoma	50	PT2977 (small molecule inhibitor of HIF2α)	Overall response rate	Recruiting	March 2023
NCT03592472	Phase 3	A Randomized, Double-blind, Placebo-controlled Study of Pazopanib with or without Abexinostat in Patients With Locally Advanced or Metastatic Renal Cell Carcinoma (RENAVIV)	413	Pazopanib + abexinostat vs. pazopanib + placebo	Progression-free survival; overall survival	Recruiting	January 2022
NCT02535533	Phase 1	A Therapeutic Trial for Safety and Preliminary Efficacy of the Combination of Axitinib and Seleniomethionine (SLM) for Adult Patients with Advanced Metastatic Clear Cell Renal Cell Carcinoma	30	SLM administrated orally twice daily for 14 days, followed by SLM once daily in combination with axitinib 5 mg twice daily	Safety	Recruiting	September 2020
NCT02974738	Phase 1	A Phase 1, Multiple-Dose, Dose-Escalation and Expansion Trial of PT2977, a HIF-2α Inhibitor, in Patients With Advanced Solid Tumors	125	PT2977	Safety	Recruiting	June 2019
NCT02293980	Phase 1	A Phase 1, Multiple-Dose, Dose-Escalation Trial of PT2385 Tablets, a HIF-2α Inhibitor, in Patients With Advanced Clear Cell Renal Cell Carcinoma	107	Part 1: PT2385 tablets; Part 2: PT2385 tablets in combination with nivolumab; Part 3: PT2385 tablets in combination with cabozantinib	Safety, DLT	Active, not recruiting	December 2018
NCT02619253	Phase 1/1b	A Phase 1/1b, Open Label, Dose Finding Study to Evaluate Safety, Pharmacodynamics and Efficacy of Pembrolizumab (MK-3475) in Combination with Vorinostat in Patients with Advanced Renal or Urothelial Cell Carcinoma	42	Pembrolizumab and vorinostat	Safety/DLT	Recruiting	May 2020
NCT03634540	Phase 2	A Phase 2 Trial of PT2977 in Combination with Cabozantinib in Patients with Advanced Clear Cell Renal Cell Carcinoma	118	PT2977 in combination with cabozantinib tablets	Overall response rate (CR, PR)	Not yet recruiting	September 2022

CR: complete response; PR: partial response; SD: stable disease; PD: progressive disease; ORR: objective response rate; OS: overall survival; N/A: not available; HIF: hypoxia inducible factor; RP2D: recommended phase II dose; RCC: renal cell carcinoma; DLT: dose-limiting toxicity.
